# ER stress dependent microparticles derived from smooth muscle cells promote endothelial dysfunction during thoracic aortic aneurysm and dissection

**DOI:** 10.1042/CS20170252

**Published:** 2017-06-07

**Authors:** Li-Xin Jia, Wen-Mei Zhang, Tao-Tao Li, Yan Liu, Chun-Mei Piao, You-Cai Ma, Yu Lu, Yuan Wang, Ting-Ting Liu, Yong-Fen Qi, Jie Du

**Affiliations:** 1 Beijing Anzhen Hospital, Capital Medical University; The Key Laboratory of Remodeling-Related Cardiovascular Diseases, Ministry of Education; Beijing Collaborative Innovation Center for Cardiovascular Disorders; Beijing Institute of Heart, Lung & Blood Vessel Disease, Beijing 100029, China; 2 Emergency & Critical Care Center, Beijing Anzhen Hospital, Capital Medical University, Beijing 100029, China; 3 Department of Biochemistry, Shanxi Medical University, Taiyuan 030001, Shanxi, China

**Keywords:** anoikis, ER stress inhibitor, inflammation, microparticles, Thoracic aortic aneurysm and dissection (TAAD)

## Abstract

The degeneration of vascular smooth muscle cell(s) (SMC) is one of the key features of thoracic aortic aneurysm and dissection (TAAD). We and others have shown that elevated endoplasmic reticulum (ER) stress causes SMC loss and TAAD formation, however, the mechanism of how SMC dysfunction contributes to intimal damage, leading to TAAD, remains to be explored. In the present study, *in vitro* assay demonstrated that elevated mechanical stretch (18% elongation, 3600 cycles/h) stimulated the ER stress response and microparticle(s) (MP) production from both SMC and endothelial cell(s) (EC) in a time-dependent manner. Treatment of EC with isolated MP led to anoikis, which was determined by measuring the fluorescence of the ethidium homodimer (EthD-1) and Calcein AM cultured in hydrogel-coated plates and control plates. MP stimulation of EC also up-regulated the mRNA levels of inflammatory molecules (i.e. Vascular cellular adhesion molecular-1 (VCAM-1)), intercellular adhesion molecular-1 (ICAM-1), interleukin-1β (IL-1β), and interleukin-6 (IL-6)). Use of an ER stress inhibitor or knockout of CHOP decreased mechanical stretch-induced MP production in SMC. *In vivo*, administration of an ER stress inhibitor or knockout of CHOP suppressed both apoptosis of EC and the infiltration of inflammatory cells. Moreover, TAAD formation was also suppressed by the administration of an ER stress inhibitor. In conclusion, our study demonstrates that elevated mechanical stretch induces MP formation in SMC leading to endothelial dysfunction, which is ER stress dependent. The inhibition of ER stress suppressed EC apoptosis, inflammation in the aorta, and TAAD development.

## Introduction

Thoracic aortic aneurysm and dissection (TAAD) accounts for approximately 10 deaths per 100000 people [1]. Both genetic factors and other risk factors contribute to TAAD formation, and although a great deal has been learned about the surgical skills, there are limited treatment options for TAAD except blood pressure control. TAAD begins with an initial tear in the aortic intima and media layers, which allows a large amount of blood to enter the media, leading to false lumen formation.

The loss of smooth muscle cell(s) (SMC) and extracellular matrix (ECM) degradation are the key features in TAAD [2]. We and others have found that mechanical stretch led to apoptosis and senescence in SMC during TAAD pathogenesis both *in vivo* and *in vitro* [3–5]. Elevated endoplasmic reticulum (ER) stress was also found to be involved in mechanical stretch induced SMC apoptosis [6,7]. However, as apoptosis of SMC is an end feature of vascular damage, there must be other vasculature abnormalities prior to apoptosis and TAAD formation. As one of the main cell types in the vasculature, it is known that endothelial cell(s) (EC) dysfunction and tearing of the intima is a critical step in TAAD formation [8]; but how EC function is impaired during TAAD formation remains unclear.

Microparticle(s) (MP) are shed from the plasma membrane and have a diameter of 0.05–1 μm. Upon physiological and pathological stimulus, MP can be produced from several cell types, including SMC [9]. In atherosclerotic plaques, MP from stressed SMC promote EC dysfunction in a β3 integrin dependent way [10]. Therefore, we hypothesized that MP originating from stressed SMC could lead to EC dysfunction, which could initiate the intimal tear in TAAD.

In the present study, we demonstrate that elevated mechanical stretch led to SMC-derived MP production in an ER stress-dependent manner, which promoted EC dysfunction and inflammation, leading to TAAD formation.

## Materials and methods

### Animal model and ethics statement

Wild-type or CHOP knockout mice of C57BL/6 background were obtained from the Jackson Laboratory (Bar Harbor, ME) and fed in a specific pathogen free (SPF) animal facility in Beijing Anzhen Hospital. The TAAD mice model was induced as described recently [3]. In brief, 3-week-old male mice were given a normal diet and administered a solution of BAPN (β-aminopropionitrile, Sigma–Aldrich, St. Louis, MO), which was dissolved in drinking water at a concentration of 1 g/kg per day for 4 weeks. BAPN treatment led to TAAD formation via inhibiting lysyl oxidase activity, which is responsible for collagen and elastin cross-linking. To assess the effect of an ER stress inhibitor, 4-phenylbutyric acid (4-PBA, Sigma) was administered via intraperitoneal injection at a concentration of 200 mg/kg per day. All studies were approved by the Institutional Animal Care and Use Committee of Beijing Anzhen Hospital, Capital Medical University, Beijing, China. The investigation conforms to the Guide for the Care and Use of Laboratory Animals published by the U.S. National Institutes of Health.

### Histology and immunohistochemistry

Histology and immunohistochemistry staining were performed as described recently [4,5]. Briefly, mouse aortas were fixed, embedded, and sectioned at 5-μm intervals, and incubated with 3% H_2_O_2_ for 10 min. Sections were then blocked with serum and incubated with primary antibodies at 4°C overnight. Primary antibodies against Mac-2 (Santa Cruz Biotechnology, CA; 1:200 dilution), Gr-1 (Abcam, Cambridge, MA; 1:200 dilution), and IgG (Santa Cruz) were used as a negative control. Sections were then incubated with the ChemMate™ EnVision™ System (Dako, Glostrup, Denmark). Images were captured and further analyzed using ImageProPlus 3.0 (ECIPSE80i/90i); additional images are provided in Supplementary Figure S1.

Cell apoptosis was detected using a commercial DeadEnd Fluorometric TUNEL Kit (Promega, Madison, WI). Costaining of Tunel with α-SMA or CD31 was performed to detect apoptotic SMC or apoptotic EC prior to confocal fluorescence microscopy analysis (Leica Microsystems, Buffalo Grove, IL).

HE staining was performed as described elsewhere. Elastin staining involved the use of an Elastic Fiber Staining Kit (Maixin Bio, Fuzhou, China) as described recently [5]. All images were further analyzed using ImageProPlus 3.0 (ECIPSE80i/90i).

### Quantitative real-time PCR

Total RNA from mouse aortas or human aortic endothelial cell (HAEC) was extracted using TRIzol reagent (Invitrogen, Carlsbad, CA), and 2 μg of RNA was reversed using the GoScript™ Reverse Transcription System (Promega) according to the manufacturer’s instructions. Quantitative real-time PCR was performed using the iQ5 system (Bio–Rad, Hercules, CA) with the primers described in Table 1. The housekeeping gene glyceraldehyde 3-phosphate dehydrogenase (*GAPDH*) was used as a control.

**Table 1:** Primers used in the present study

Gene names	Organism	Forward	Reverse
*IL-1β*	Mouse	5′-CTTCAGGCAGGCAGTATCACTCAT-3′	5′-TCTAATGGGAACGTCACACACCAG-3′
*IL-6*	Mouse	5′-CTTCCATCCAGTTGCCTTCTTG-3′	5′-AATTAAGCCTCCGACTTGTGAAG-3′
*TNF-α*	Mouse	5′-GCCACCACGCTCTTCTGTCT-3′	5′-GTCTGGGCCATGGAACTGAT-3′
*Atf4*	Mouse	5′-CCTATAAAGGCTTGCGGCCA-3′	5′-GCTGGATTTCGTGAAGAGCG-3′
*GRP78*	Mouse	5′-TACTGGCCGAGACAACACTG-3′	5′-TACTGGCCGAGACAACACTG-3′
*CHOP*	Mouse	5′-CTTGAGCCTAACACGTCGATT-3′	5′-CCAGGTTCTCTCTCCTCAGGT-3′
*GAPDH*	Mouse	5′-CCTGGAGAAACCTGCCAAGTATGA-3′	5′-TTGAAGTCACAGGAGACAACCTGG-3′
*VCAM-1*	Human	5′-CCGTCTCATTGACTTGCAGC-3′	5′-GATGTGGTCCCCTCATTCGT-3′
*ICAM-1*	Human	5′-AGGATGGCACTTTCCCACTG-3′	5′-GGAGAGCACATTCACGGTCA-3′
*IL-1β*	Human	5′-TGAGCTCGCCAGTGAAATGA -3′	5′-AGATTCGTAGCTGGATGCCG -3′
*IL-6*	Human	5′-TCAATATTAGAGTCTCAACCCCCA-3′	5′-TTCTCTTTCGTTCCCGGTGG -3′
*Atf4*	Human	5′-GGGAAGCGATTTAACGAGCG-3′	5′-CTACGCTTTCCCGATCCCAG-3′
*GRP78*	Human	5′-GAACGTCTGATTGGCGATGC-3′	5′-TCAAAGACCGTGTTCTCGGG-3′
*CHOP*	Human	5′-TTCTCTGGCTTGGCTGACTG-3′	5′-TCCTCCTCTTCCTCCTGAGC-3′
*GAPDH*	Human	5′-GGTTGTCTCCTGCGACTTCA-3′	5′-GGTGGTCCAGGGTTTCTTACTC-3′

ICAM-1, intercellular adhesion molecular-1; IL-1β, interleukin-1β; IL-6, interleukin-6; VCAM-1, vascular cellular adhesion molecular-1.

### SMC culture, cyclic stretch, and flow cytometry

SMC were isolated from mouse aorta as previously described [11]. In brief, the mouse aorta was separated and digested with type II collagenase at 37°C for 30 min to remove the adventitia. The mouse aorta was then digested with a mixture of collagenase and elastase for 30 min after removing the endothelium by gently rubbing the intimae. SMC were cultured in an SMC medium (SMCM; Sciencell, Carlsbad, CA) containing 10% FBS, 1% penicillin-streptomycin (PS), and 1% SMC growth supplement (SMCGS). We chose the *in vitro* model, in which SMC were subjected to elevated mechanical stretch (18% elongation) to partially mimic *in vivo* weakening of the aorta in pathological dissection conditions. The *in vitro* model was realized using the Flexcell system (Flexcell 5000), which can apply a gradient biaxial strain of up to 33% elongation (Flexcell Inc. Corporation, Hillsborough, NC). As for the degree of elongation, it is reported that low magnitude stretches of 5–10% are considered to be a physiological stretch, whereas high magnitude stretches of 20% and above are considered to be a pathological stretch, such as hypertension [12,13]. SMC were cultured on silicone, elastomer bottomed, collagen-coated plates (Flexcell Inc. Corporation, Hillsborough, NC) and were subjected to cyclic mechanical stretch using a computer controlled mechanical strain unit (Flexcell 5000) at a condition of 18% elongation, as recently described [4]. To inhibit ER stress, 1 mM 4-PBA was administered 30 min before the mechanical stretch.

MP which had been subjected to mechanical stretch were isolated from the SMC medium. The conditioned medium was collected and centrifuged at 1500×***g*** for 10 min to clear the cells and debris. The supernatant was then further centrifuged at 12500×***g*** for 1 h at 10°C. After being washed twice, MP were suspended in DMEM, and stored at –80°C until use.

The MP isolated from the SMC medium were counted by flow cytometry with the antibody PE–conjugated anti-Annexin-V (BD Bioscience, San Jose, CA, U.S.A.) and 2-μm beads as described before [10,14]. In brief, MP (10 μl) were resuspended in binding buffer and incubated with the FITC–conjugated Annexin V (eBioscience, San Diego, CA) for 15 min at room temperature in the dark, and then washed twice with PBS. Before flow cytometry analysis, 2 µm sized beads (Spherotech, Lake Forest, IL, U.S.A.) were used to define the events. A total of 10000 events were analyzed. In addition, polystyrene beads of defined sizes (0.46 and 1 µm) were used to provide a size guide.

### Anoikis assay

EC anoikis was detected using a CytoSelect™ 96-Well Anoikis Assay kit (Cell Biolab) according to the manufacturer’s protocol [15–17]. The kit allows the quantitation and monitoring of anoikis in cells using a precoated hydrogel plate. The assay principal is based on the fact that the hydrogel-coated plate was anchorage resistant for cells, while the non-coated plate was used as the control. Live cells were viewed under a microscope and quantitated on a plate reader by MTT (colorimetric) or Calcein AM (fluorometric); dead cells were detected using red ethidium homodimer (EthD-1). In brief, primary HAEC (ScienCell) were plated on a control plate and a hydrogel-precoated plate and cultured in EC medium (ScienCell) supplemented with 5% FBS and 1% EC growth factor. After a 48-h incubation period at 37°C, the stimulated MP were isolated from the SMC medium after being stretched for 48 h, EthD-1 was added into each well to detect apoptotic cells and Calcein AM was added into each well to detect live cells. The plates were incubated for 30–60 min at 37°C. The EthD-1 and Calcein AM content of each well was determined with a Synergy HD plate reader (BioTek, Vermont, U.S.A.) and Gen5 software (BioTek, Vermont, U.S.A.), using 525/590 and 485/515 nm filters for excitation and emission, respectively. Images were captured with a Leica microscope (M165FC) conjugated to a Leica digital color camera (DFC310FX, Leica Microsystems Inc., Buffalo Grove, IL), and acquired with LASv4.1 imaging software (Leica Microsystems Inc.).

### Statistical analysis

In all the cases, results from at least three independent experiments were used to calculate the mean ± S.E.M. Data analysis involved the use of GraphPad Prism 5.00 for Windows. For multiple group comparison, one-way ANOVA was conducted across all the groups first, and post hoc pairwise tests for two groups, with assessment of statistical significance performed after Bonferroni correction of the overall significance level. For comparison between two groups, the Student’s *t* test was conducted. *P*<0.05 was considered statistically significant.

## Results

### Mechanical stretch induced MP production from SMC promoting HAEC dysfunction

MP are small particles released from multiple cells when subjected to physiological or pathological stimuli [9]. We previously set up a mouse model of TAAD by administering BAPN; as is shown in Supplementary Figure S2, while the diameters of mouse ascending aortas did not show a difference either with vehicle or BAPN treatment, the diameter of the aortic arch increased in the BAPN-administered group compared with that in the vehicle group 4 weeks after administration. Moreover, the *V*_max_ in the ascending aorta and aortic arch were also higher after BAPN administration for 4 weeks compared with the vehicle group.

**Figure 1:**
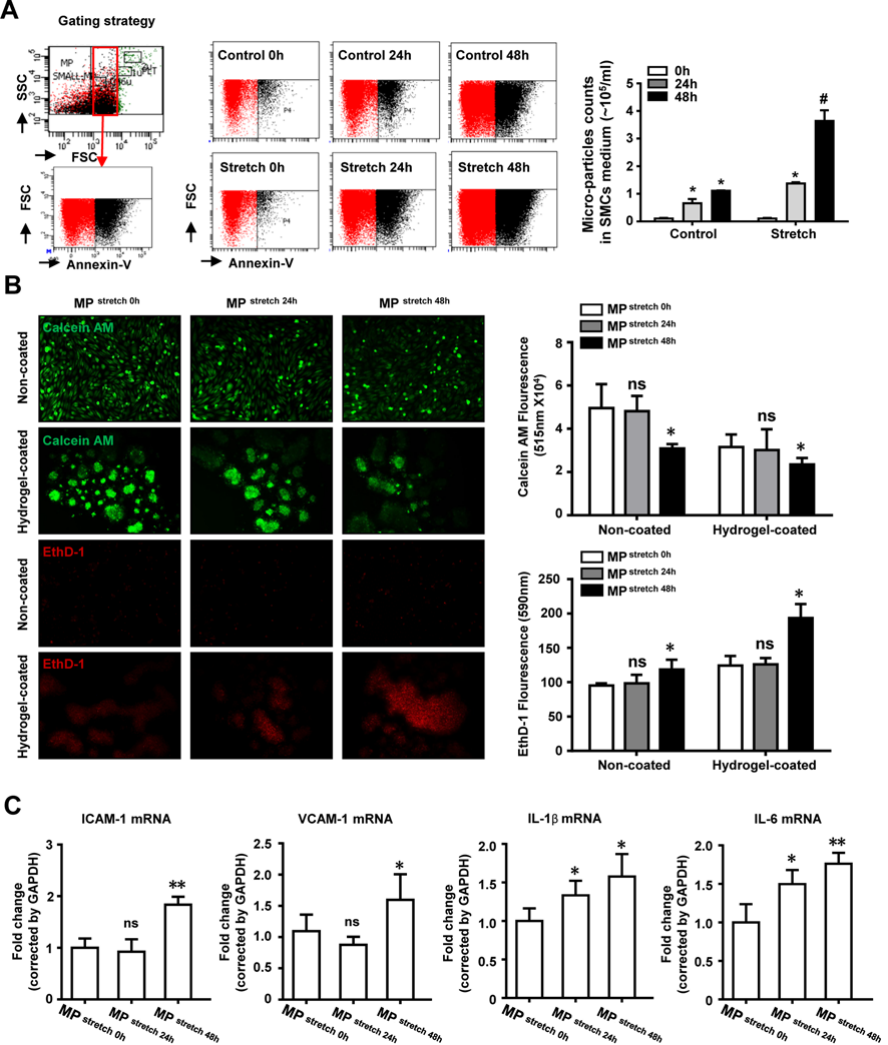
SMC-derived MP in response to mechanical stretch promotes HAEC dysfunction (**A**) Flow cytometry analysis of MP production in cultured SMC medium at the indicated time either in static conditions or after being stretched. (**B**) Representative pictures of fluorescence of Calcein AM or EthD-1 in HAEC stimulated with MP for 48h. (**C**) Real-time PCR analysis showing the mRNA levels of ICAM-1, VCAM-1, IL-1β, and IL-6 in HAEC after stimulation with MP for 48 h. *n*=3 in each group, *P<0.05, **P<0.01, compared with control group; ns, not significant.

To examine whether elevated mechanical stretch could induce MP production, we treated cultured SMC with mechanical stretch (18% elongation, 3600 cycles/h). The medium was then collected at the indicated time, and MP counts were quantitated by flow cytometry. MP produced from cultured SMC were stretched or not and measured by flow cytometry at the indicated time. As shown in Figure 1A, SMC produced MP under basal conditions, while mechanical stretch significantly increased MP production. To explore the role of MP, cultured HAEC were stimulated with the SMC medium after being stretched. The CytoSelect™ 96-Well Anoikis Assay Kit was then used to detect HAEC anoikis. As is shown in Figure 1B, after being stretched for 48 h, MP from SMC significantly increased the amount of apoptotic HAEC plated in both anchorage resistant and control plates. The mRNA levels of adhesion molecules and pro-inflammatory cytokines were also measured using real-time PCR, while the levels of ICAM-1, VCAM-1, IL-6, and IL-1β were all up-regulated in HAEC after being stimulated with MP (Figure 1C).

### ER stress inhibitor decreased mechanical stretch induced MP production and HAEC dysfunction

We and others have reported that ER stress is involved in mechanical stretch induced SMC apoptosis and TAAD formation, we thus examined whether MP production is ER stress dependent. The mRNA levels of ER stress-related genes (*GRP78, ATF4*, or *CHOP*) in aortas at day 0, 7, 14, and 28 after BAPN administration were examined using quantitative real-time PCR (Supplementary Figure S3A), and all these genes were found to be up-regulated. These gene and protein levels were also evaluated in human TAAD specimens and normal aortas. As shown in Supplementary Figure S3B,C, RT-PCR and immunohistostaining showed that the expression of ER stress-related molecules ATF4, GRP78, and CHOP were elevated in human TAAD specimens compared with that in the normal aorta.

**Figure 2:**
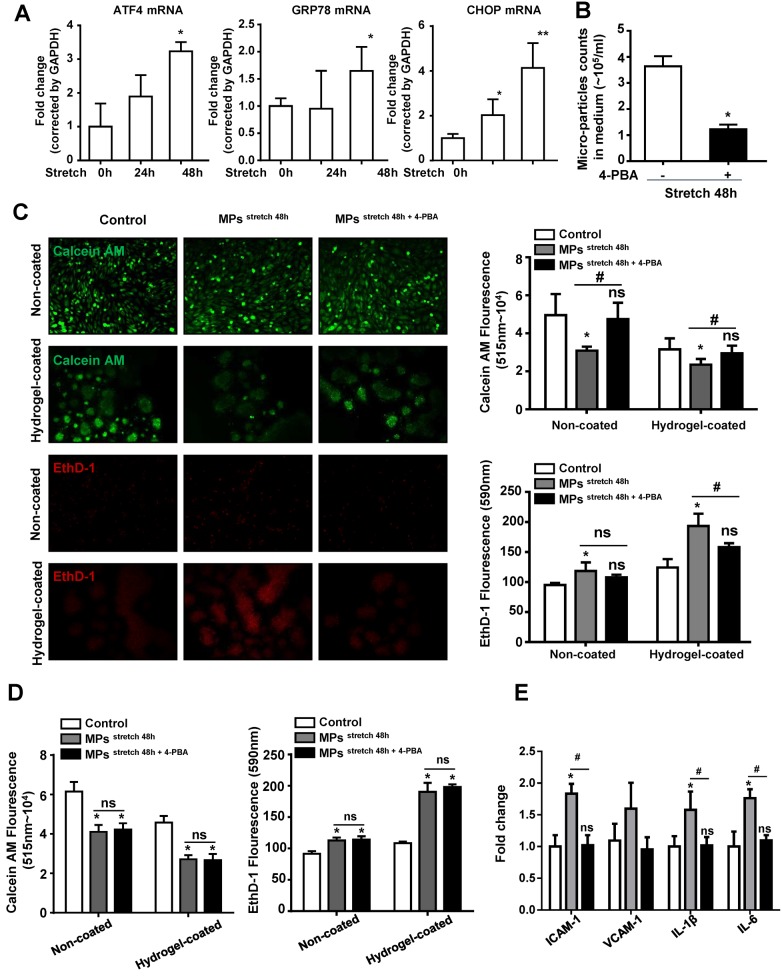
Stretch-induced MP production and HAEC dysfunction is ER stress dependent (**A**) Real-time PCR analysis shows the mRNA levels of GRP78, ATF4, and CHOP in SMC after being stretched at the indicated time, *n*=3 in each group, *P<0.05, compared with control group; ns, not significant. (**B**) Flow cytometry analysis of MP production from SMC after being stretched for 48 h with or without 4-PBA, *n*=3 in each group, *P<0.05, compared with the -4-PBA group. (**C**) Representative pictures and fluorescence of Calcein AM or EthD-1 in HAEC after stimulation with MP for 48 h, and MP were isolated from the same volume medium of SMC after being stretched for 48 h with or without 4-PBA. (**D**) Bar graph showing fluorescence of Calcein AM or EthD-1 in HAEC after stimulation with MP for 48 h, and isolated MP from the SMC medium after being stretched 48 h with or without 4-PBA were adjusted to the same quantity. (**E**) Real-time PCR analysis showing the mRNA levels of ICAM-1, VCAM-1, IL-1β, and IL-6 in HAEC after being stimulated with MP for 48 h. *n*=3 in each group, *P<0.05, compared with control group, ^#^*P*<0.05, ^##^*P*<0.01, compared with stimulated MP but no 4-PBA treatment; ns, not significant.

The mRNA levels of ER stress-related genes were also measured in SMC after being subjected to mechanical stretch. The data show that the expressions of GRP78, ATF4, and CHOP were up-regulated in SMC after being stretched (Figure 2A). We therefore treated SMC with an ER stress inhibitor (4-PBA), 30 min before being stretched. Flow cytometry analysis showed that 4-PBA reduced MP production from SMC after being stretched for 48 h (Figure 2B). Furthermore, the anoikis assay of HAEC showed that the conditional medium from 4-PBA-treated SMC failed to induce apoptosis of HAEC (Figure 2C). To examine if the effect of an *in vitro* assay is dependent on the MP concentration, MP from the cultured SMC, after being stretched with or without 4-PBA treatment, was adjusted to an equal number and added to the HEAC. In this case, there was no significant difference in anoikis between the two groups, thus the observed effect is quantity dependent (Figure 2D). 4-PBA treatment also inhibited the increase in mRNA levels of ICAM-1, IL-1β, and IL-6 up-regulated by SMC-derived MP without 4-PBA treatment (Figure 2E).

In addition to the role of VSMC-derived MP, whether mechanical stretch could induce MP generation from cultured HAEC was also examined. Similar to the results of SMC, HAEC produced MP under either basal or stretch conditions, and HAEC-derived MP significantly increased after being stretched for 48 h (Supplementary Figure S4A). Treatment with the ER stress inhibitor 4-PBA not only decreased MP generation from HAEC after being stretched for 48 h, but also showed a protective role in MP-induced HAEC anoikis (Supplementary Figure S4B,C).

### ER stress inhibitor suppresses BAPN-induced TAAD formation

**Figure 3:**
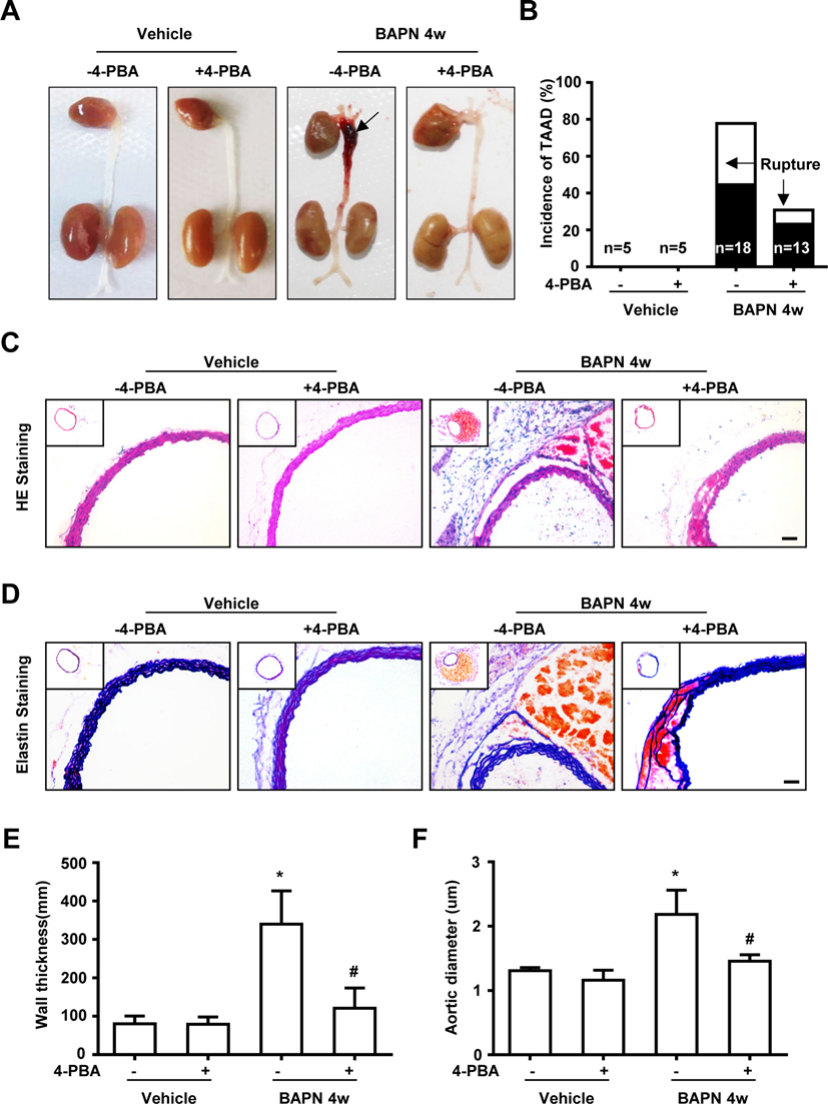
ER stress inhibitor suppresses BAPN administration induced TAAD formation (**A**) Representative photos show macroscopic features of mouse aortas with or without 4-PBA after administration with the vehicle or BAPN for 28 days, arrow indicates the TAAD. (**B**) Incidence and rupture of TAAD after BAPN treatment with or without 4-PBA. Representative H E staining (**C**) and elastin staining, (**D**) of aortas from mice with or without 4-PBA after administration with the vehicle and BAPN. Bar graph shows the wall thickness, (**E**) and aortic diameter, (**F**) of thoracic aorta. Data are mean ± S.E.M. from three experiments. *P<0.05, compared with vehicle; ^#^*P*<0.05, compared with BAPN administration without 4-PBA.

To further evaluate the role of ER stress in TAAD pathogenesis, we treated mice with 4-PBA via intraperitoneal injection. The representative pictures showed that BAPN administration resulted in TAAD formation, the arrow shows the big thromboci; while 4-PBA treatment or CHOP knockout suppressed TAAD formation. The bar graph shows the statistical analysis of the ratio of TAAD formation and rupture (confirmed by autopsy), and 4-PBA treatment suppressed not only TAAD development, but also TAAD rupture (Figure 3A&B). HE staining and elastin staining were also performed to show that the pathological features of either inflammatory cell infiltration or elastin degradation was inhibited by administering 4-PBA in BAPN-induced TAAD formation (Figure 3C,D). Further analysis of wall thickness and aortic dimeter showed similar results (Figure 3E,F).

### 4-PBA treatment decreased EC apoptosis as well as inflammation in BAPN-induced TAAD mouse

**Figure 4:**
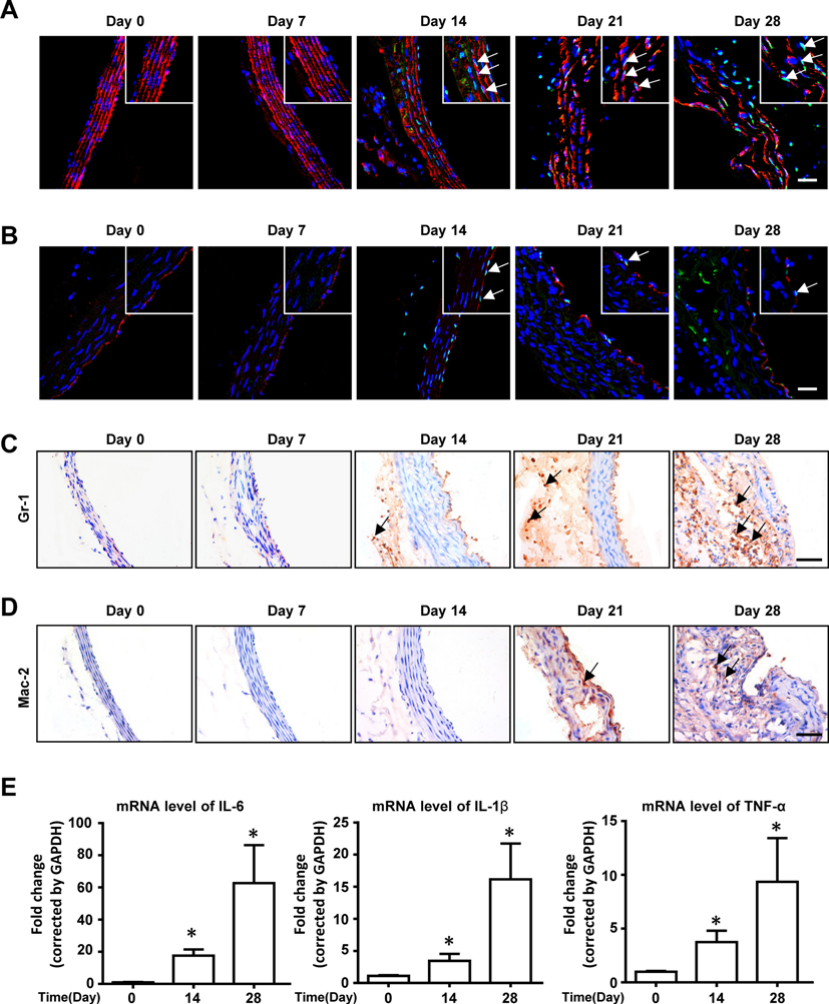
Apoptosis and inflammation are involved in BAPN administration induced TAAD formation Representative pictures of costaining of TUNEL with α-SMA (**A**), and Tunel with CD31 (**B**) in aortas at indicated times after BAPN administration, white arrows indicate double positive cells. Representative pictures of immunohistochemical staining for (**C**) Gr-1 and (**D**) Mac-2 in mouse aortas at indicated times after BAPN administration, black arrows indicated positive cells. (**E**) Real-time PCR analysis shows the mRNA levels of IL-1β, IL-6, and TNF-α in mouse aortas at indicated times after BAPN administration. *n*=4 in each group, *P<0.05, compared with time zero.

We and others have reported that cell apoptosis, as well as inflammation, play a key role in TAAD formation. Inhibition of inflammatory cell infiltration [18] or cytokine production [19] suppressed aortic aneurysm and dissection formation. We thus performed TUNEL staining in mouse aortas after BAPN administration. As is shown in Figure 4A, costaining of TUNEL and α-SMA showed that SMC apoptosis appeared at day 14 after BAPN administration. EC apoptosis, defined by TUNEL and CD31 double positive cells, also showed a similar result (Figure 4B). Moreover, inflammatory cell infiltration was also detected by immunohistostaining. Gr-1 staining showed that accumulated neutrophils in both the intima and adventitial appeared at day 14 after BAPN treatment, while Mac-2 staining showed macrophage infiltration at day 21 (Figure 4C,D). Real-time PCR analysis showed that the mRNA levels of inflammatory cytokines in mouse aortas, including IL-6, IL-1β, and TNF-α, were also up-regulated after BAPN administration (Figure 4E).

**Figure 5:**
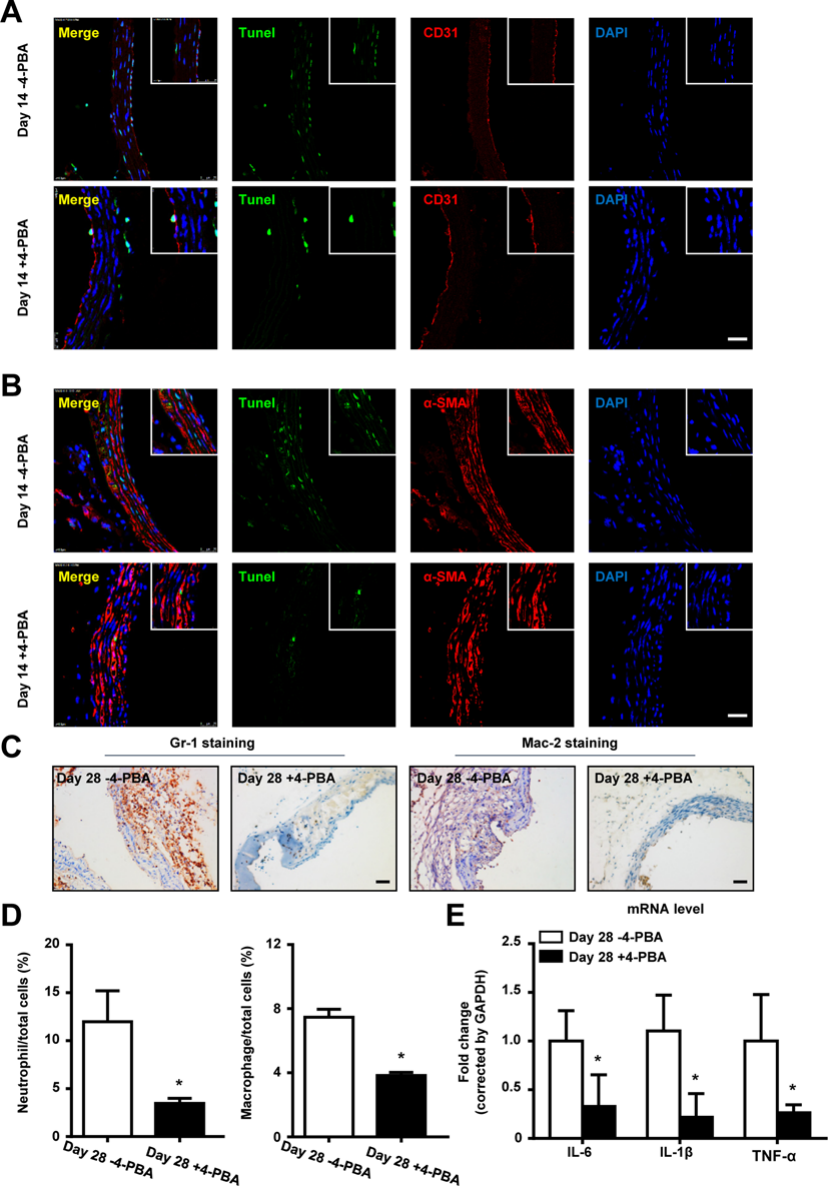
4-PBA treatment decreases EC apoptosis as well as inflammation in BAPN administration induced TAAD mice Representative pictures of costaining of TUNEL with (**A**) α-SMA and (**B**) CD31 in mouse aortas at day 14 after BAPN administration with or without 4-PBA treatment. (**C**) Representative pictures of immunohistochemical staining for Gr-1 and Mac-2 in mouse aortas at day 28 after BAPN administration with or without 4-PBA. (**D**) Bar graph showing the percentage of infiltrated neutrophils and macrophages in aortas after BAPN administration for 28 days with or without 4-PBA treatment. (**E**) Real-time PCR analysis showing the mRNA levels of IL-1β, IL-6, and TNF-α in mouse aortas after BAPN administration for 28 days with or without 4-PBA treatment. *n*=4 in each group, *P<0.05, compared with -4-PBA group.

To determine if the treatment with an ER stress inhibitor decreased EC apoptosis, costaining of TUNEL and CD31 in BAPN-treated mice aortas, which had been exposed to an ER stress inhibitor, was performed. EC apoptosis was inhibited upon 4-PBA administration, although SMC apoptosis was also suppressed (Figure 5A,B). *In vitro*, 4-PBA treatment also decreased mechanical stretch induced SMC and HAEC apoptosis (Supplementary Figure S5). Moreover, neutrophils and macrophages infiltrated BAPN-treated mouse aortas with or without 4-PBA treatment. As shown in Figure 5C,D, Gr-1^+^ neutrophils and Mac-2^+^ macrophages accumulated in BAPN-treated mouse aortas, while 4-PBA treatment decreased the infiltration of these inflammatory cells. Moreover, the mRNA levels of IL-1β, IL-6, and TNF-α, detected by real-time PCR, were all up-regulated in response to BAPN administration, which was inhibited by 4-PBA treatment (Figure 5E).

## Discussion

The present study reports for the first time that mechanical stretch induced MP production by both SMC and EC is ER stress dependent, which leads to EC dysfunction and contributes to TAAD formation. Moreover, an ER stress inhibitor or CHOP knockout (Supplementary Figure S6) not only blocks MP production *in vitro*, but also suppresses BAPN-induced TAAD formation and rupture, thus, an ER stress inhibitor could be a potential treatment of TAAD.

MP are small particles which are released after cell activation or apoptosis [20], and can be generated from either circulating cells or cells present in vessel walls. Although the specific mechanisms that lead to MP formation have not been completely elucidated, MP are effectors capable of delivering biological messages to target cells [21]. It is reported that MP mediate intercellular communication. [22,23]. In the present study, MP were isolated from the culture medium after the same amount of SMC had been stretched with or without 4-PBA treatment. The anoikis assay showed the protective role of 4-PBA, which could be the result of the difference in amount or characteristics of MP. Indeed, 4-PBA treatment reduced the production of MP. To explore the possibility, we adjusted the number of MP from SMC being stretched with or without 4-PBA treatment to the same quantity. In this case, there was no significant difference in anoikis between the two groups (Figure 2D). Taken together, the observed effect of 4-PBA resulted from it reducing the production of MP. Moreover, the stimulus that triggered MP formation determined the composition of MP and, consequently, the biological information that they transfer [24,25]. In the present study, we showed that elevated mechanical stretch is an important and physiological relevant stimulus to induce MP production from vascular SMC, and BAPN stimulation showed no effect on MP production and apoptosis in both SMC and HAEC *in vitro* (Supplementary Figure S7). In a normal aorta, SMC are aligned in the media of the artery, and subjected to mechanical stretch via pulsatile blood flow. Mechanical stretch was found to modulate SMC alignment, differentiation, migration survival/apoptosis as well as its secretion [26] through activating intracellular signaling pathways, including JNK [6], Rho-associated kinase/ROCK, NF-κB-inducing kinase [27], MAPK/ERK kinase (MEKK) [28] etc. Importantly, mechanical stretch induced MP production is ER stress dependent as we have previously shown that elevated mechanical stretch induces apoptosis of SMC in an ER stress-dependent fashion [4]. Our current study further identifies that MP production is also associated with these events, and MP from apoptotic SMC could be messengers causing vascular damage. Indeed, an *in vitro* study had shown that mechanical stress-induced TNF-α secretion from cultured rat SMC modulated CHOP expression and mediated SMC apoptosis [6]. It should be noted that MP are produced by both VSMC and EC, it is therefore possible that the MP generated from EC could affect the processes involved.

Anoikis is defined as the programmed cell death by loss of connection to the correct ECM, thus disrupting integrin ligation [29]. It is known that SMC loss and ECM degradation are two key features of TAAD pathogenesis. Several groups have explored the role of MP on EC function. MP from human atherosclerosis increased both EC proliferation and stimulated *in vivo* angiogenesis, thereby accelerating the progression of atherosclerotic lesions by promoting intraplaque neovascularization and increasing their thrombogenicity [30]. Circulating MP from patients after myocardial infarction (MI) impaired the endothelial nitric oxide transduction pathway, which contributed to the vascular dysfunction after MI in rats [31]. Moreover, MP from cultured human T cells also induced endothelial dysfunction through regulation of the protein expression for endothelial NO synthase and caveolin-1 [32]. In the present study, our results show that both SMC and EC-derived MP cause EC dysfunction by inducing the anoikis and inflammation of EC. These results extend our understanding of how stressed SMC induce EC dysfunction. To further determine the cellular source of MP *in vivo*, we also measured the production of circulating MP, including total MP (Annexin-V^+^), EC-derived MP (Annexin-V^+^CD31^+^), platelet-derived MP (Annexin-V^+^CD41^+^), and leukocyte-derived MP (Annexin-V^+^CD45^+^). The results show that these MP increased 4 weeks after BAPN administration, while 4-PBA treatment inhibited the increase in circulating MP (Supplementary Figure S8). Taken together, we have shown that there were increases in MP production in a mouse model of TAAD and that 4-PBA treatment suppressed this effect. Along with our results from the mechanically stretched model of SMC for *in vitro* study, it would be ideal to demonstrate that most MP *in vivo* are from SMC. However, there is a technical limitation to determining the quantity of MP derived from SMC *in vivo* due to current lack of specific surface markers for SMC.

We recently reported that deletion of CHOP, a key transcriptional factor regulating ER stress mediated apoptosis, prevented SMC loss and TAAD formation [4]. These results suggested that inhibition of ER stress could be a potential therapeutic target. In the present study, we have shown that administration of an ER stress inhibitor (4-PBA) suppressed SMC apoptosis and TAAD development (Figures 3 and 5), which strongly suggests that an ER stress inhibitor could be a pharmacological target for TAAD. Our results are consistent with several studies, 4-PBA, a ‘chemical chaperone’, has been demonstrated to improve the misfolding of proteins in ER stress, thereby inhibiting ER stress in response to both physiological and pathophysiological stimuli [33]. Moreover, 4-PBA is now used in clinical trials for sickle-cell anemia [34] and β-thalassemia [35], and is useful in cystic fibrosis patients [36].

In conclusion, our present study demonstrates that mechanical stretch induced MP production from SMC is ER stress dependent, and contributes to TAAD formation through stimulating EC dysfunction. The ER stress inhibitor 4-PBA could be a potential therapeutic target for TAAD formation and progression. The present study used an ER stress inhibitor as a preventive strategy prior to an aneurysm developing. One limitation of our present study is that the appearance of this pathological condition is unpredictable and once it develops, it is too late for the use of drugs (e.g. 4-PBA).

## Clinical perspectives

- Vascular SMC loss is one of the key features of TAAD. We and others showed that elevated ER stress causes SMC loss and TAAD formation, however, mechanism whereby how SMC dysfunction contribute to intimal damage, leading to TAAD remaining to be explored.
- Our study demonstrated that elevated mechanical stretch induced MP formation in SMC leading to endothelial dysfunction, which is ER stress dependent. ER stress inhibition suppressed ECs apoptosis, inflammation in aorta, and TAAD development.
- ER stress inhibitor could be a potential treatment target of TAAD.

## Abbreviations

Atf-4: activating transcription factor-4
BAPN: β-aminopropionitrile
CHOP: C/EBP homologous protein
DMEM: Dulbecco minimum essential medium
EC: endothelial cell
ECM: extracellular matrix
ER: endoplasmic reticulum
ERK: extracellular regulated protein kinases
EthD-1: ethidium homodimer
GAPDH: glyceraldehyde 3-phosphate dehydrogenase
GRP78: glucose-regulated protein 78
HAEC: human aortic endothelial cell
HE: hematoxylin-eosin
ICAM-1: intercellular adhesion molecular-1
IL-1β: interleukin-1β
IL-6: interleukin-6
JNK: c-Jun N-terminal kinase
MAPK: mitogen-activated protein kinase
MI: myocardial infarction
MP: microparticle
NF-κB: nuclear factor kappa B
ROCK: Rho-associated coiled-coil kinase
SMC: smooth muscle cell
TAAD: thoracic aortic aneurysm and dissection
TNF-α: tumor necrosis factor-α
TUNEL: terminal deoxynucleotidyl transferase-mediated deoxyuridine triphosphate-biotin nick end labeling
VCAM-1: vascular cellular adhesion molecular-1
4-PBA: 4-phenylbutyric acid
